# Calculating trauma triage precision: effects of different definitions of major trauma

**DOI:** 10.1186/1752-2897-6-9

**Published:** 2012-08-17

**Authors:** Hans Morten Lossius, Marius Rehn, Kjell E Tjosevik, Torsten Eken

**Affiliations:** 1 Department of Research and Development, The Norwegian Air Ambulance Foundation, Holterveien 24, PO Box 94, NO-1441 Drøbak, Norway; 2 Field of Pre-hospital Critical Care, Network of Medical Sciences, University of Stavanger, Kjell Arholmsgate 41, NO-4036 Stavanger, Norway; 3 Department of Anaesthesia and Intensive Care, Akershus University Hospital, Sykehusveien 25, NO-1478, Lørenskog, Norway; 4 Acute Clinic, Stavanger University Hospital, Armauer Hansens vei 20, NO-4011, Stavanger, Norway; 5 Department of Anaesthesiology, Oslo University Hospital Ullevål, Kirkeveien 166, NO-0450, Oslo, Norway

## Abstract

**Background:**

Triage is the process of classifying patients according to injury severity and determining the priority for further treatment. Although the term “major trauma” represents the reference against which over- and undertriage rates are calculated, its definition is inconsistent in the current literature. This study aimed to investigate the effects of different definitions of major trauma on the calculation of perceived over- and undertriage rates in a Norwegian trauma cohort.

**Methods:**

We performed a retrospective analysis of patients included in the trauma registry of a primary, referral trauma centre. Two “traditional” definitions were developed based on anatomical injury severity scores (ISS >15 and NISS >15), one “extended” definition was based on outcome (30-day mortality) and mechanism of injury (proximal penetrating injury), one ”extensive” definition was based on the “extended” definition and on ICU resource consumption (admitted to the ICU for >2 days and/or transferred intubated out of the hospital in ≤2 days), and an additional four definitions were based on combinations of the first four.

**Results:**

There were no significant differences in the perceived under- and overtriage rates between the two “traditional” definitions (NISS >15 and ISS >15). Adding “extended” and “extensive” to the “traditional” definitions also did not significantly alter perceived under- and overtriage. Defining major trauma only in terms of the mechanism of injury and mortality, with or without ICU resource consumption (the “extended” and “extensive” groups), drastically increased the perceived overtriage rates.

**Conclusion:**

Although the proportion of patients who were defined as having sustained major trauma increased when NISS-based definitions were substituted for ISS-based definitions, the outcomes of the triage precision calculations did not differ significantly between the two scales. Additionally, expanding the purely anatomic definition of major trauma by including proximal penetrating injury, 30-day mortality, ICU LOS greater than 2 days and transferred intubated out of the hospital at ≤2 days did not significantly influence the perceived triage precision. We recommend that triage precision calculations should include anatomical injury scaling according to NISS. To further enhance comparability of trauma triage calculations, researchers should establish a consensus on a uniform definition of major trauma.

## Background

Early appreciation of major trauma enables emergency medical service (EMS) providers to match the available resources to each victim’s needs. Triage is the process of classifying patients according to injury severity and determining the priority for further treatment [1,2]. Field triage has become increasingly important, as regionalised trauma care with dedicated trauma teams has been shown to improve patient outcome [3-5]. Nevertheless, some mistriage is unavoidable, as field triage is performed close to the time of injury, with limited diagnostic resources in a multifarious pre-hospital environment. If major trauma victims are undertriaged and therefore denied access to high-resource resuscitation, avoidable negative outcomes may ensue [1,6]. Conversely, overtriage may cause minor trauma victims to be unnecessarily transferred to dedicated trauma care facilities, thereby consuming scarce financial and human resources. Overtriage thus decreases the available resources for other patients with greater needs [7,8].

The rates of over- and undertriage are considered to be trauma system quality indicators [2]. Although these data are debated, the American College of Surgeons - Committee on Trauma (ACS-COT) states that an undertriage rate of 5–10% is unavoidable, and most systems are associated with an overtriage rate of 30–50% [2,9].

The definition of major trauma provides the reference standard against which the over- and undertriage rates are calculated [10]. There is a 40-year tradition of grading the severity of individual injuries using the Abbreviated Injury Scale (AIS), and based on this scale, the Injury Severity Score (ISS) can be calculated as the sum of the squares of the highest AIS code in each of the three most severely injured ISS body regions [11,12]. The US Major Trauma Outcome Study (MTOS) found that an ISS >15 was associated with a mortality risk of at least 10% and was related to a distinct increase in mortality [13]. Following this study, many subsequent triage studies dichotomised study populations into “major trauma” patients, who were defined as having an ISS >15, and “minor and moderate trauma” patients (ISS ≤15), and they presented two-by-two tables describing the diagnostic accuracy of triage algorithms [10,14]. Several limitations of the ISS have been highlighted [15,16], providing a basis for the New Injury Severity Score (NISS) [17]. The NISS is a simple modification of the ISS and is calculated from the three most severe injuries regardless of body region. The NISS has been considered to be more predictive of survival, especially in patients suffering from multiple head injuries or penetrating trauma [17-20]. Although the ISS is still the dominant scale in papers published on triage precision, an NISS >15 is recommended as an inclusion criterion in the Utstein template for uniform reporting of data following major trauma [21].

Mortality and morbidity are the principal outcomes after trauma, and their relevance remains undisputed [22,23]. To define major trauma, several studies have therefore combined anatomic injury scales, such as the ISS or NISS, with variables associated with mortality, morbidity, type of injury, or resource consumption [24-26]. The rationale is an understanding of major trauma as more complex than anatomic injury alone. The compound definitions in these studies often include process-mapping variables, making the definitions more system-specific and thereby reducing the external validity and reproducibility.

Butcher et al. reported, in their review on the definitions of “polytrauma”, that there was no consensus on the term [27]. This lack of consensus was corroborated by a recent systematic review of pre-hospital prognostic trauma models [28], in which the authors also questioned the external validity of published studies on triage precision and emphasised the challenges inherent in the comparison of triage systems.

To compare data sets, assess external validity and facilitate multicentre trials, the impact of different definitions of major trauma on quality assessments should be clarified. The aim of the present study was to investigate how various definitions of major trauma influence the calculation of under- and overtriage in a trauma cohort.

## Methods

### Study population

Stavanger University Hospital (SUH) is a 630-bed hospital and is the primary trauma centre for a mixed rural/urban population of approximately 330,000 inhabitants. It is also the trauma referral centre for all 440,000 people living in Rogaland County in southwestern Norway. SUH admits approximately 140 adult and paediatric patients annually with NISS scores >15 and treats approximately 3,400 patients per year with minor injuries [26]. During the study period, the hospital practised the informal activation of a one-tiered, 13-personnel, large, multidisciplinary trauma team. Pre-hospital emergency care in the catchment area was provided by on call general practitioners, ground ambulance units staffed with paramedics, and anaesthesiologist-staffed rapid response cars and helicopters.

### Study design

Since 2004, a hospital-based trauma registry has been fully operational. An Association for the Advancement of Automotive Medicine certified AIS coder (a registered nurse) manually searches the hospital administrative data system for eligible patients and annually codes the data from approximately 360 individuals (see Table 1 for the trauma registry inclusion and exclusion criteria).

**Table 1:** Inclusion and exclusion criteria for the Stavanger University Hospital (SUH) trauma registry

**Inclusion Criteria**	**Exclusion Criteria**
*Absolute:*	*Patients only fulfilling relative criteria are excluded if:*
· Activated trauma team	
· Penetrating injury to:	· Isolated fracture and skin injury (AIS 1) in:
∘ Head	∘ Upper extremity
∘ Neck	∘ Lower extremity
∘ Trunk	∘ Floor of the orbit
∘ Extremities proximal to the knee or elbow	· Chronic subdural haematoma
	· Drowning, inhalation injury, asphyxia-related injury (hanging, strangulation)
*Relative:*	
· ISS ≥10	· Secondary admission to SUH >24 hours after injury

ISS: Injury Severity Score; AIS: Abbreviated Injury Scale.

We performed a retrospective analysis of the SUH trauma registry data and included consecutive patients who were admitted to SUH between January 1, 2004, and December 31, 2008, and had been assigned one or more AIS codes (AIS 98; Abbreviated Injury Scale, 1990 Revision, Update 98). Inter-hospital transfers to SUH and patients transported by non-healthcare personnel were excluded, as they were not subject to SUH field triage practices. Survival status 30 days after injury was obtained from the Norwegian Population Registry and from patient records [21].

The Regional Committee for Medical and Health Research Ethics deemed their formal approval unnecessary (2009/228-CAG). The Norwegian Social Science Data Services approved our access to anonymous data from relevant patients in the trauma registry (20840 KS/LR).

### Definitions of major trauma

We constructed eight different definitions of major trauma (Table 2). Both ISS >15 and NISS >15 have been recommended as definitions of major trauma, and therefore two of the definitions were based solely on these anatomic injury severity scores.

**Table 2:** Definitions of “major trauma” used in the study

**ISS traditional**	**ISS >15**
ISS extended	“ISS traditional” **and/or** “Dead 30 days after injury” **and/or** “Proximal penetrating injury”
ISS extensive	“ISS extended” **and/or** “ICU LOS >2 days” **and/or** “ICU LOS ≤2 days and transferred out intubated”
NISS traditional	NISS >15
NISS extended	“NISS traditional” **and/or** “Dead 30 days after injury” **and/or** “Proximal penetrating injury”
NISS extensive	“NISS extended” **and/or** “ICU LOS >2 days” **and/or** “ICU LOS ≤2 days and transferred out intubated”
Extended	“Dead 30 days after injury” **and/or** “Proximal penetrating injury”
Extensive	“Extended” **and/or** “ICU LOS >2 days” **and/or** “ICU LOS ≤2 days and transferred out intubated”

ISS = Injury Severity Score; NISS = New Injury Severity Score; ICU = intensive care unit; LOS = length of stay.

It has been argued that trauma resulting in patient death must be considered as major [24-26]. Further, penetrating trauma to vital body structures results in rapid deterioration and death if not treated urgently, and this provides a rationale for defining proximal penetrating injury as major trauma. Therefore, one “extended” definition based on death within 30 days and/or proximal penetrating injury was added. Additionally, one “extensive” definition based on the “extended” definition combined with high resource consumption was constructed. Based on available variables in our registry, we defined high resource consumption as intensive care of more than two days. As some SUH patients are transferred to other hospitals during ongoing intensive care due to need for specialised care or limited ICU capacity, both "admitted to the ICU for >2 days" and "transferred intubated out of the hospital at ≤2 days" were included in this definition. Four additional definitions were constructed from combinations of the original definitions (Table 2). Perceived triage precision was calculated according to each of these eight separate definitions of major trauma.

### Statistical analysis

The calculation of perceived triage precision was based on the assumption that all of the patients suffering from major trauma, according to the above definitions, should have access to the trauma team upon hospital admission. Undertriage rate was defined as the proportion of patients who were not triaged to a trauma team despite having a major trauma (c/(a + c) in Table 3), i.e., the complement of the sensitivity (1-sensitivity) [25]. Overtriage rate was defined as the proportion of patients without major trauma among those who were triaged to a trauma team (b/(a + b) in Table 3), i.e., the complement of the positive predictive value (1-PPV), where PPV denotes the probability that a patient suffers from a major trauma when the trauma team is activated. The 95% confidence intervals (95% CI) for over- and undertriage were calculated as $p\pm 1.96\times \sqrt{\frac{p\times \left(1-p\right)}{n}}$, where *p* is the proportion of patients that had been over- or undertriaged and *n* is the total number of patients who were triaged by a trauma team or had experienced major trauma (a + b and a + c in Table 3). Significant differences were defined as non-overlapping 95% confidence intervals.

**Table 3:** Injury severity and trauma team activation (TTA)

	**Major trauma**	**Non-major trauma**	**Total**
**TTA**	a	b	a + b
**No TTA**	c	d	c + d
**Total**	a + c	b + d	n

Sensitivity = a/(a + c); Specificity = d/(b + d); Positive predictive value (PPV) = a/(a + b).

Undertriage = 1-Sensitivity = c/(a + c); Overtriage = 1-PPV = b/(a + b).

## Results

### Descriptive

**Figure 1:**
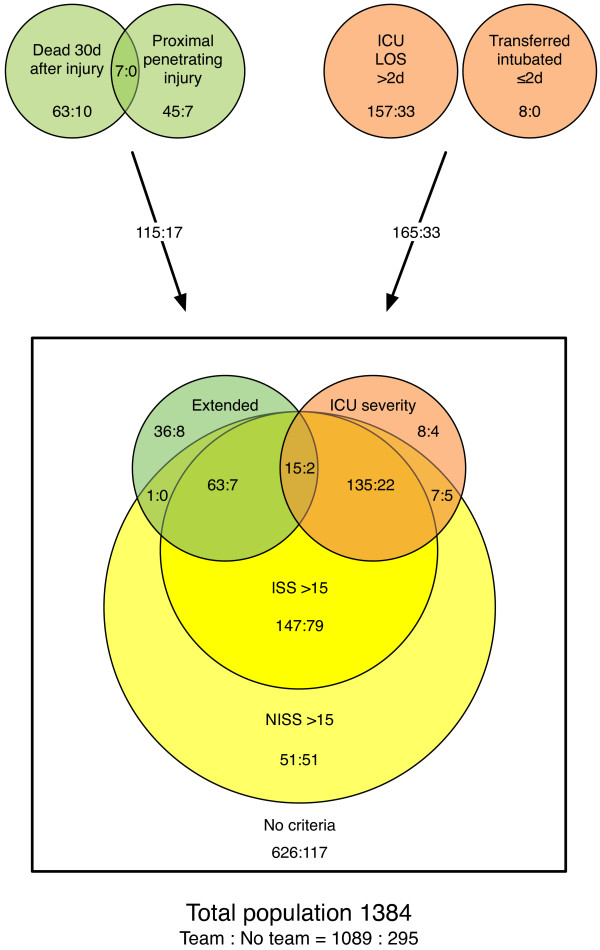
**Set diagram of definitions for major trauma (circles); overlapping areas represent patients covered by two or more definitions.** The “extensive” definition used in our study consisted of both “extended” and “ICU severity”. The number of patients triaged to be received by a trauma team is provided together with the number of patients not met by a team.

**Figure 2:**
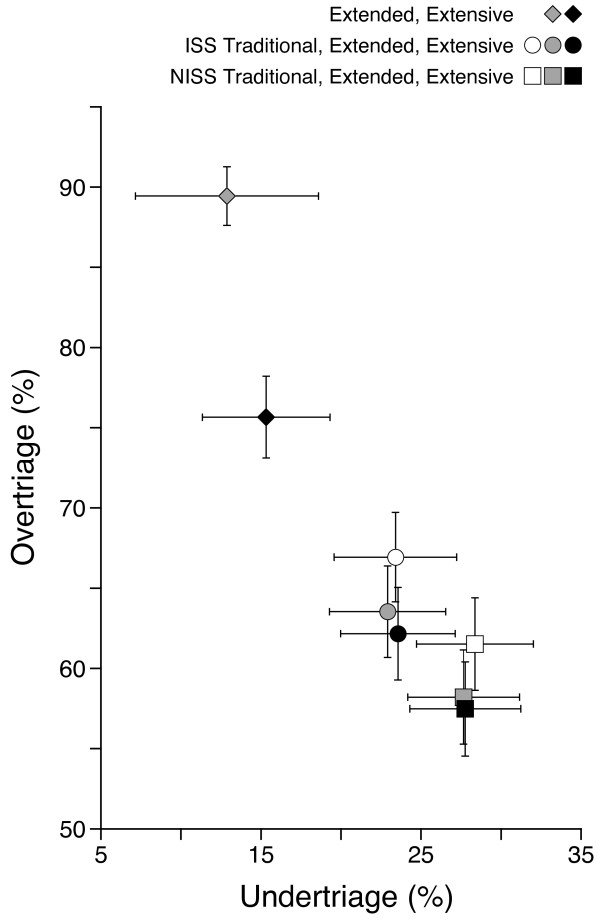
**Consequences of the various definitions of major trauma for perceived over- and undertriage.** ISS-based definitions are shown as circles, NISS-based definitions are shown as squares, and diamonds represent definitions that are not based on anatomic criteria (cf. Tables 3 and 4). The symbols representing “extended” and “extensive” definitions are grey and black, respectively. The lines denote 95% confidence intervals.

During the study period, of the 1 481 patients who were coded in the SUH trauma registry, 1 384 fulfilled our eligibility criteria (cf. Table 1 and Figure 1). Among these included patients, 1 315 (95%) suffered blunt injuries, and 69 (5%) suffered penetrating injuries. The median age was 31 years old (IQR 19–51), and 997 (72%) of the patients were male. The median ISS score was 10 (IQR 5–19), the median NISS score was 12 (IQR 5–24), and 80 patients died within 30 days (mortality 5.8%). Figure 1 shows the number of patients falling within the combinations of the various definitions and highlights the proportion of patients who were met by a trauma team. There was a significant increase in the percentage of patients who were defined as having sustained a major trauma when the NISS-based definitions were compared to their ISS-based counterparts (p < 0.01 for all comparisons). Table 4 shows the proportions of the included patients having sustained a major trauma according to the various definitions, with the corresponding values for over- and undertriage (see also Figure 2).

**Table 4:** Number and proportions of included patients with major trauma according to the different definitions and perceived triage precision

**Definition of major trauma**	**Number of major trauma patients (% of total population)**	**Perceived undertriage (%) with 95% CI**	**Perceived overtriage (%) with 95% CI**
ISS Traditional	470 (34.0)	23.4 (19.6 – 27.2)	66.9 (64.1 – 69.7)
ISS Extended	515 (37.2)	22.9 (19.3 – 26.5)	63.5 (60.7 – 66.4)
ISS Extensive	539 (38.9)	23.6 (20.0 – 27.1)	62.2 (59.3 – 65.0)
NISS Traditional	585 (42.3)	28.4 (24.7 – 32.0)	61.5 (58.6 – 64.4)
NISS Extended	629 (45.4)	27.7 (24.2 – 31.2)	58.2 (55.3 – 61.1)
NISS Extensive	641 (46.3)	27.8 (24.3 – 31.2)	57.5 (54.5 – 60.4)
Extended	132 (9.5)	12.9 (7.2 – 18.6)	89.4 (87.6 – 91.3)
Extensive	313 (22.6)	15.3 (11.3 – 19.3)	75.7 (73.1 – 78.2)

### Triage quality assessment

There were no significant differences between perceived under- and overtriage using NISS >15 or ISS >15, NISS “extended” or ISS “extended”, or NISS “extensive” or ISS “extensive” as definitions of major trauma, except for the ISS Traditional definition (i.e., ISS >15), which had higher perceived overtriage than NISS “extended” and NISS “extensive” (Table 4 and Figure 2). The major trauma definitions without anatomic criteria, i.e., “extended” (based on type of injury and 30-day survival only) or “extensive” (“extended” combined with ICU LOS >2 days or transferred out intubated within 2 days), resulted in significantly lower perceived undertriage than the NISS-based definitions and significantly higher perceived overtriage than any other definition (Table 4 and Figure 2).

## Discussion

The definition of major trauma is commonly based on anatomic injury alone, and both ISS >15 and NISS >15 are recommended cut-off values. Our study revealed no significant differences in the perceived under- and overtriage rates between NISS >15 or ISS >15 as the definitions for major trauma. This finding suggests that the outcomes of triage precision calculations may be comparable between trauma systems, regardless of the use of NISS >15 or ISS >15 as definitions. In contrast, the NISS will be equal to or greater than the ISS for any given patient, depending on the injuries sustained. Accordingly, utilising NISS >15 instead of ISS >15 will result in an increased number of included patients in most trauma populations (cf. Table 4 and Figure 1). In the present population, we found a 24% relative increase in the number of patients who were defined as having sustained a major trauma when NISS >15 was applied, compared to ISS >15 (from 470 to 585; see Table 4). This increase might be interpreted as improved sensitivity without loss of specificity, implying that NISS >15 is superior to ISS >15 as a definition of major trauma [18,29]. However, this difference in sensitivity caused by the use of a different injury scale obviously makes the results less comparable. It has therefore been argued that a compound definition of major trauma is necessary [25]. Factors other than anatomic injury influence outcome, and the inclusion of the mechanism of injury and/or outcome variables, such as mortality, in the definition of major trauma seems relevant. The Utstein template recommends 30-day mortality, Glasgow Outcome Scale (GOS), discharge destination, and hospital length of stay (LOS) to be reported as outcome measurements after trauma [21]. In an attempt to capture the complexity of trauma, several studies have included such outcomes in their definitions of major trauma [6,25,30-32]. In our cohort, expanding the purely anatomic definition of major trauma by including proximal penetrating injury, 30-day mortality, ICU LOS greater than 2 days and transferred intubated out of the hospital at ≤2 days did not significantly influence the perceived triage precision.

Defining major trauma only in terms of the mechanism of injury, death within 30 days and resource consumption, without including anatomic injury scaling, drastically reduced the number of cases defined as major trauma. The proportions of perceived major trauma patients in the study population were reduced from 34.0% and 42.3% with ISS >15 and NISS >15, respectively, to 9.5% in the “extended” group and 22.6% in the “extensive” group (Table 4), thereby putting into serious doubt the usefulness of these definitions for triage precision calculations.

### Limitations

The present study presents a fairly small amount of data from a single centre, and its findings are dependent on the SUH trauma population’s characteristics, including a very low number of penetrating injuries. The findings may also be susceptible to bias caused by idiosyncrasies of the informal trauma triage system at SUH. Thus, applicability to other trauma populations could be limited. Furthermore, the retrospective nature of this study restricted the data to variables that were already defined and coded in the institutional trauma registry.

## Conclusion

The definition of major trauma provides a reference standard when calculating the precision of trauma triage. However, the definitions are inconsistent in the current literature. In our cohort, although the proportion of patients who were defined as having sustained major trauma increased when NISS-based definitions were substituted for ISS-based definitions, the outcomes of the triage precision calculations did not differ significantly between the two scales. Additionally, adding the mechanism of injury and outcome variables did not significantly influence the triage precision calculations. Based on our findings we recommend that triage precision calculations should include anatomical injury scaling according to NISS. To further enhance comparability of trauma triage calculations, researchers should establish a consensus on a uniform definition of major trauma.

## Competing interest

The authors declare that they have no conflicts of interests regarding this study.

## Authors’ contributions

HML and MR conceived and planned the study. TE designed and built the SUH trauma registry, KET included and coded all of the patients, KET and MR extracted the data, and TE performed the analyses. All of the authors participated in the drafting and completion of the manuscript. All authors read and approved the final manuscript.
